# Revolutionising Quality Management in the Oral Pathology Laboratory: A Deep Dive Into the Six Sigma Methodology

**DOI:** 10.7759/cureus.52651

**Published:** 2024-01-21

**Authors:** Neha Kannan, Karthikeyan Ramalingam, Pratibha Ramani

**Affiliations:** 1 Oral Pathology and Microbiology, Saveetha Dental College and Hospitals, Saveetha Institute of Medical and Technical Sciences, Saveetha University, Chennai, IND

**Keywords:** total quality management, quality indicator, quality management system, clinical laboratory quality management, quality improvement tool, quality management, oral pathology laboratory, lean six sigma, lean methods, six sigma

## Abstract

Six Sigma Foundations is a statistical standard that indicates an exceptionally high level of quality, along with a customer satisfaction management approach that intends to lower error rates and boost process efficiency. The Define, Measure, Analyse, Improve, and Control (DMAIC) approach is a fundamental component of Six Sigma and provides an organised framework for process improvement. In contrast to conventional techniques that are more manual-based, Six Sigma emphasises and focuses on making decisions based on facts and evidence. The key to the success of Six Sigma is its reliance on statistical methods. Advanced tools like Pareto charts, histograms, regression analysis, and fishbone diagrams are used ardently for the benefit of customers and to reduce the overall error rate. To support clinical decision-making, a clinical laboratory's primary responsibility is to generate test results that are accurate, repeatable, fast, and appropriately interpreted. Ensuring desired clinical outcomes must be the ultimate objective. To accomplish this goal, laboratories must prioritise cost-effectiveness while establishing and maintaining quality in all laboratory procedures. The concept of the Lean Six Sigma (LSS) methodology, which mainly centres on efficiency by discerning and eradicating actions or operations that do not provide any benefit to the organisation, is combined with the proposition of Six Sigma, which emphasises data-driven analyses and optimization. The integration of these powerful concepts aids in the overall improvement of the organisations adopting these techniques. This review provides a brief overview of the benefits of the LSS methodology and its implementation in the oral pathology laboratory.

## Introduction and background

Six Sigma is a quality and customer satisfaction management approach that intends to lower error rates and boost process efficiency [1]. Since Motorola began using it in the mid-1980s, it has evolved into a powerful management tool that aims to achieve a 99.99996% quality level [2]. Organisations in a variety of industries are using Six Sigma, a potent approach that has completely changed the field of quality management, in their never-ending quest for excellence. Though it has evolved from its manufacturing roots, Six Sigma is now a standard for anybody looking to reduce errors, streamline workflows, and improve lab operations as a whole [3].

Oral pathology is essential for accurate diagnosis, early detection of oral diseases, informed treatment planning, research and development, and linking oral health to overall health. It plays a vital role in maintaining good oral hygiene, preventing serious health problems, and improving the quality of life for individuals. Lab management is not just a set of protocols; it's a philosophy that prioritises quality, safety, efficiency, and compliance. By prioritising these aspects, labs can ensure the accuracy of their work, protect their personnel and the environment, and contribute meaningfully to their respective fields. The medical literature regarding effective lab management in oral pathology is scarce. In this review, we have attempted to stress the implications of the Six Sigma methodology in the management of oral pathology laboratories.

## Review

Understanding the essence of Six Sigma

The Statistical Benchmark

The Six Sigma Foundation is a statistical standard that indicates an exceptionally high level of quality (3.4 failures per million opportunities). With the implementation of this strict standard, firms will be able to improve their processes methodically and usher in a new era of accuracy and consistency [4].

The Define, Measure, Analyze, Improve, and Control (DMAIC) Methodology

The DMAIC approach is a fundamental component of Six Sigma and provides an organised framework for process improvement [5].

The “Define” Phase

The project goal and the team's scope must be precisely defined by the healthcare organisation's top management during the Define phase per the agreement. Senior management needs to communicate with the team about "what the project is and what it needs to achieve" to understand the client's needs [5]. The Define phase is where project objectives and client needs are outlined. The analysis of the project aim and its length is, in fact, the primary responsibility of the define phase. Attestation of the team's and management's mutual understanding is done at this stage. The organisation often establishes the project boundaries while creating a process map by marking the beginning and finishing locations on the project map [6].

Implications in the Oral Pathology Laboratory

**Figure 1:**
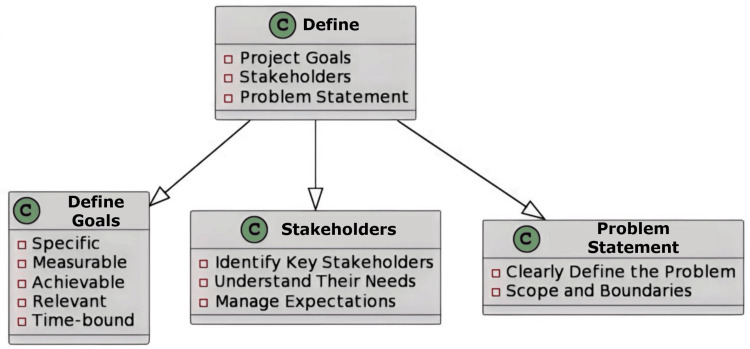
An illustration of the Define phase in lab management Image credit: Neha Kannan and Karthikeyan Ramalingam

This stage clearly states what the oral pathology laboratory's goals are and determines the important procedures, like sample gathering, analysis, reporting, and recognising the needs of customers, including timely report delivery and compliance with regulations (Figure 1).

The “Measure” Phase

The phases that follow include measuring the performance of the current process, identifying the underlying causes, putting improvements into place, and setting up safeguards to keep things moving forward. The operational concept of critical quality (CTQ) is used in this step to identify the measurable service indicators. Along with identifying the input and outcome measurements, this phase also establishes baseline metrics, systematically arranges array data in visual representations, and adheres to statistical rigour by assembling and summarising [7]. After the measurement has taken into account the voice of customers (VOC), the consumer feedback is converted into quantifiable design needs. For individuals used to DMAIC projects, the focus on VOC could be unfamiliar. The VOC analysis highlights that a precise grasp of customer demands is a critical success factor, even though the needs of the customer determine the priorities of a DMAIC project. The gathering and analysis of data ought to involve the project managers. To measure the needs of the customers, they should ask the members of the team to determine what kinds of data should be collected, why, and how to use it [7, 8]. The Ishikawa diagram, also called the cause-and-effect diagram, can be used in this stage to note different potential gaps in routine processes [8].

Implications in the Oral Pathology Laboratory

**Figure 2:**
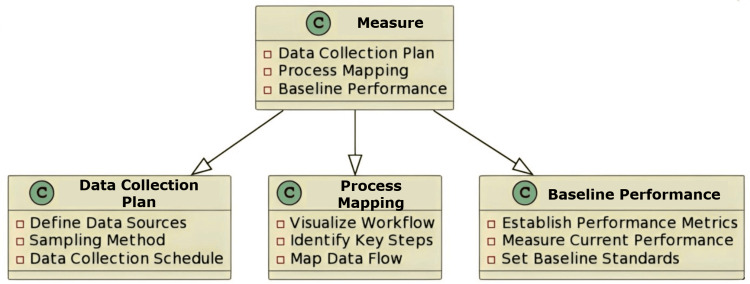
An illustration of the Measure phase in lab management Image credit: Neha Kannan and Karthikeyan Ramalingam

It helps in creating performance measures for the laboratory, such as diagnosis accuracy and report turnaround time, and provides methods for gathering data with an emphasis on pivotal moments in the various processes, as a result of which it identifies the state of the processes as they stand to find opportunities for improvement (Figure 2).

The “Analyze” phase

This stage examines the gathered information and applies a value stream map (VSM) to determine and confirm the root causes of errors that have an impact on the non-value-added (NVA) procedures. This phase examines the data gathered during the measurement phase to pinpoint the origin of waste, delays, and subpar performance. The team finds issues with the service processes during the Analyze phase. This stage, which stresses using a data-driven and perspective-based approach to decision-making, is a crucial way that Six Sigma differs from other methodologies. A skilled person can spot issues with service procedures and lead a group through a VSM event to figure out what needs to be done to make value-added and non-voluntary work for customers [9]. It is essential to carefully document all of the findings and ideas throughout the analysis phase. This thorough documentation is essential since it serves as the foundation for the improvement phase that follows. The knowledge acquired through analysis provides a strong basis for well-informed decision-making and focused improvements along the path of continuous improvement [9].

Implications in the Oral Pathology Laboratory

**Figure 3:**
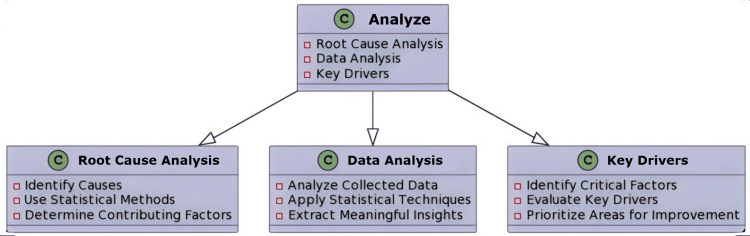
An illustration of the Analyze phase in lab management Image credit: Neha Kannan and Karthikeyan Ramalingam

Organising the data from the information gathered will enable the creation of a comprehensive picture of the current state of affairs and the interplay of many elements [10]. Finding the causes of problems like delayed reporting and mislabeling of specimens, along with inefficiencies in the process, is the main objective of the Analyze phase, which allows the project team to focus on resolving the most pressing issues first. It analyses data on sample processing, possible mistakes, and diagnostic accuracy using statistical techniques. The group can then modify the project charter as needed (Figure 3).

The “Improve” Phase

The project team addresses the underlying issues that have affected the standard of the regular procedures throughout this phase. By removing errors, waste, and expenses associated with meeting customer needs determined in the Define phase, the Improve phase modifies the service processes [11]. This stage chooses the finest options to satisfy the needs of the clients using standard tools and techniques. The team must employ the solution matrices tool to opt for the most appropriate options because it is related to ideation, the goal of the project, and ways to satisfy client needs. After deciding which options best meet the demands of the client, the project manager should focus on the implementation procedures. In this phase, the major focus shifts from analysis to implementation. To evaluate suggested solutions on a smaller scale and enable modifications for full-scale adoption, pilot testing is often used. The data collected from the pilot studies can be documented for further use and execution in larger areas. For constant improvements, these documented data will provide a strong foundation [12].

Implications in the Oral Pathology Laboratory

**Figure 4:**
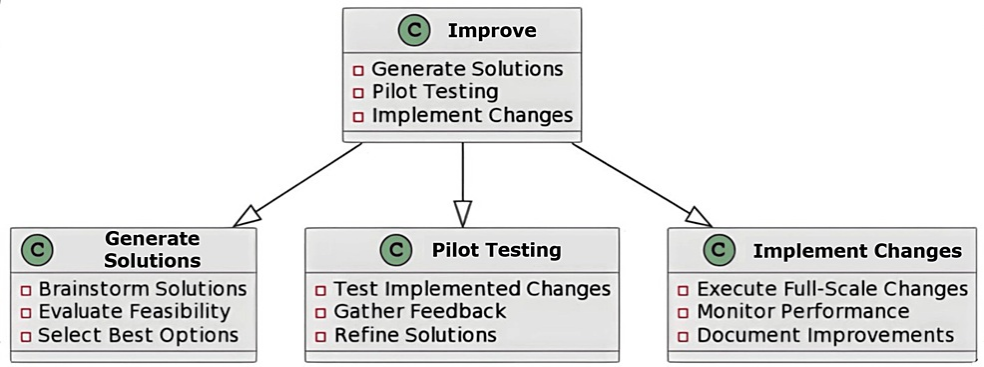
An illustration of the Improve phase in lab management Image credit: Neha Kannan and Karthikeyan Ramalingam

The main goal of the Improve phase is to implement modifications to address the challenges identified and enhance the procedure. In a pathology laboratory setting, techniques to improve the reporting period and prevent delayed reporting can be achieved by implementing various strategies. It aids in creating and executing changes to the workflow for pathology. Implementing training initiatives for lab personnel to improve proficiency and reduce error rates can be useful [11] (Figure 4).

The “Control” phase

The goal of the Control phase is to find long-term fixes. To do this, it is necessary to monitor and manage the factors that are essential to process performance. When they return to work, the project team should make sure that everyone is using the same updated procedures by imparting their knowledge to others who will be taking over. By designating who is in charge of what in the new process, a control process plan typically expands upon the future state process map. This phase emphasises sustaining the improvements that were made during the previous phases to ensure long-term success. To detect any deviations from the standard protocol set, various statistical roots are used. These tools monitor whether each step is being implemented correctly and accurately as per the newly designed protocol [13].

Implications in the Oral Pathology Laboratory

**Figure 5:**
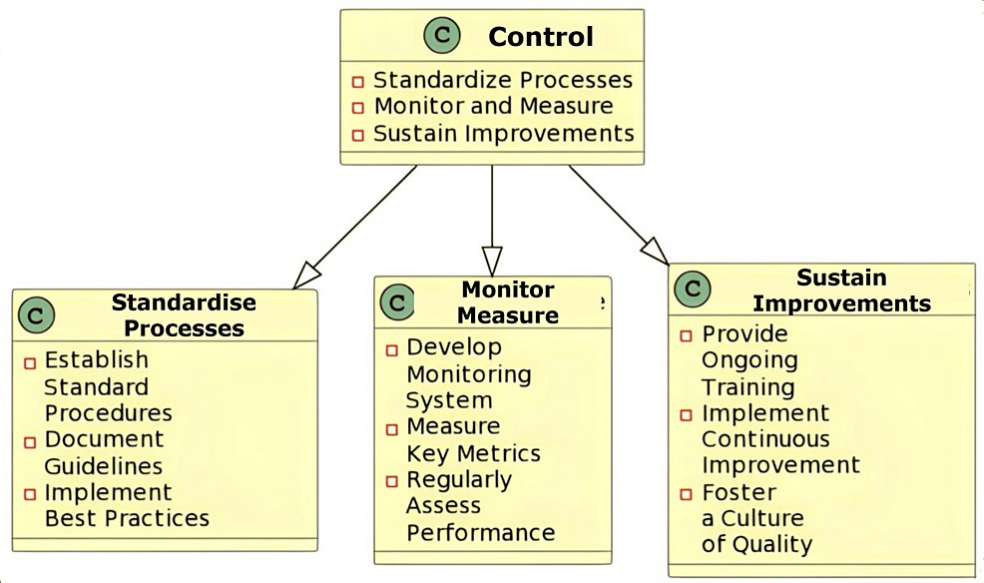
An illustration of the Control phase in lab management Image credit: Neha Kannan and Karthikeyan Ramalingam

This step ensures that the improvements made are maintained over time. It is at this stage that controls or regulations must be designed and implemented to monitor the process and prevent regression or backward movements. Individuals should be assigned to control each sector of the laboratory. Storage and retention of pathological slides and blocks, monitoring the concentrations of the solutions or chemicals used for processing and decalcification, preparation of stains, and maintaining the records should be delegated to specific individuals. To keep pathology services at a consistent level of quality, ongoing training and assistance should be given persistently. Thus, it establishes continuing key performance indicator monitoring systems [14] (Figure 5).

The influence of data-based decision-making

A Transition from Intuition to Evidence

Clinical laboratories are extremely dynamic, complicated companies that constantly need to follow strict requirements, enhance test quality, and aim to reduce expenditures. In contrast to conventional techniques that are more manual-based, Six Sigma emphasises and focuses on making decisions based on facts and evidence [15]. Various statistical tools and approaches enable the laboratory to effectively traverse complicated difficulties. This is done by providing them with a detailed, in-depth understanding of pertinent information [16].

Emphasis on Statistical Techniques 

The key to the success of Six Sigma is its reliance on statistical methods. Advanced tools like Pareto charts, histograms, regression analysis, and fishbone diagrams are used ardently for the benefit of customers and to reduce the overall error rate [16]. Reducing the error rate through the use of artificial intelligence in a laboratory setting can enhance the outcome and have many other benefits, which are listed below.

Benefits 

Customer Satisfaction and Loyalty

The good quality achieved as a result of using Six Sigma increases customer satisfaction and thereby heightens the reputation of the organization. This is achieved by using various tools like feedback analysis and process mapping, which aid in meeting customer satisfaction levels by meeting their needs [17]. Overall, this will result in increased customer retention and positive word-of-mouth improving the total revenue of the organization. Essentially, the interplay among customer happiness, loyalty, and Six Sigma guarantees a systematic strategy for improving client experiences and loyalty and maintaining business success via ongoing enhancement [16].

Expenditure Management 

The implementation of Six Sigma in the laboratory gives a balanced and great expenditure management with good efficiency [17]. Six Sigma analyses the various expenditures at every phase by using different tools and provides input on where the costs can be cut without compromising on quality. It helps in recognising and assessing any financial uncertainties through an in-depth examination of the possible negative impacts that process modifications might have on the financial aspects of an organization. Managing these risks is crucial for making financial-altering decisions that will ensure stability and prevent unwanted expenditures [18].

Leadership's Role

High-level dedication and knowledge of the laboratory in-charge and personnel are also needed for a successful workflow, along with the enactment of the Six Sigma strategy [18]. To obtain an effective workflow along with the application of Six Sigma, substantial dedication and expertise from both the laboratory in-charge and personnel and active participation from both sides are required. The laboratory leadership should possess a profound comprehension of Six Sigma methodologies to ensure successful implementation and guide the team through the different phases of the DMAIC cycle.

Applications of Lean Six Sigma (LSS) in an oral pathology laboratory

To support clinical decision-making, a clinical laboratory's primary responsibility is to generate test results that are accurate, repeatable, fast, and appropriately interpreted. Ensuring desired clinical outcomes must be the ultimate objective. Laboratories must prioritise cost-effectiveness while establishing and maintaining quality in all laboratory procedures to accomplish this goal [19].

Clinical laboratories now have to manage growing workloads with a wider range of parameters with the same or limited number of employees while still producing findings that are high-quality, consistent, and have better turnaround times (TATs). Over 70% of important medical decisions, including patient admission, treatment, and discharge, are influenced by laboratories, despite accounting for less than 5% of healthcare expenditures. However, there is a growing demand for clinical laboratories to save expenses without sacrificing or even raising quality requirements [20].

There must be constant efforts made to make costs reasonable to support the continual expansive growth of diagnostic laboratory testing within the limits of a limited budget [21]. Eliminating "waste" from the analytical phase as well as the pre-and post-analytical phases would simplify the overall laboratory procedure and help to accomplish this goal. Lean is a quality-improvement methodology that emphasises delivering “value” and enhancing performance through the methodical elimination of waste, which we characterise as anything that detracts from the end product or service [20]. To create suitable solutions for the laboratory, it is essential to have an in-depth grasp of every step of the sample collection, transportation to the laboratory, sample preparation, analytical methods, post-analytic sample handling, and validation of the results process [22].

The concept of Lean, which mainly centres on efficiency by discerning and eradicating actions or operations that do not provide any benefit to the organisation, is combined with the proposition of Six Sigma, which emphasises data-driven analyses and optimization. The integration of these powerful concepts aids in the overall improvement of the organisations adopting these techniques [23]. Following the implementation of the Lean approach in their laboratory, Morón-Castañeda et al. conducted before-and-after research that showed an improvement in patient satisfaction and a decrease in the number of complaints related to delays [24].

Management strategies should focus on changing the underlying causes of changes rather than just the specific task components that are changed because these causes are frequently poorly understood and require further research. Several research studies have examined the significance of Lean in anatomy and surgical pathology labs for raising patient and physician satisfaction, decreasing TATs, and enhancing the precision of diagnostic and molecular tests [25, 26]. Smith et al concluded in another study that addressing flawed anatomic pathology workflow processes may have a direct bearing on modifying the variables that enhance patient safety [26].

According to research by Inal et al., three hours of non-value-adding labour were removed, which improved the pre-analytical process in the waiting area of the reception. Sample turnaround times decreased from 68 to 59 minutes with the application of Lean. Steps that could cause medical mistakes and put receptionists at risk for biological risks were cut from 30% to 3%. There exist further accounts of the use of Lean concepts leading to favourable results, such as decreased operational costs and enhanced work-life equilibrium for laboratory staff [27].

In a study by Gijo et al., the waiting time for patients for sample collection was calculated. The project team determined that "the patient waiting time for sample collection" would be defined as the period from the token's issuance until the patient departed the counter. The "waiting time for sample collection" decreased from 23.96 minutes to 11 minutes as a result of the initiative [28]. Laboratory information system (LIS) implementation and modifications have an impact on TAT [29].

Ibrahim et al [30] used LSS to improve the timeliness of clinical laboratory test results in a university hospital in Egypt and demonstrated the use of LSS to successfully improve the timeliness of inpatient routine CBC tests, the primary cause of customer dissatisfaction, at the hematology laboratory. Tosuner et al emphasized LSS applications in a multicenter pathology laboratory and reported that LSS shortened the sampling time and laboratory process time of the pathology specimens, and the establishment of standards in the pathology laboratory saved considerable working time [31].

## Conclusions

To sum up, laboratory management must prioritise quality to save expenses, boost productivity, and enhance user satisfaction. To successfully apply the DMAIC and Lean models, a healthcare organisation must secure the commitment and cooperation of specialists. By using these two strategies, the healthcare organisation will be able to analyse the overall patient safety, financial results, satisfaction of the patients, and their loyalty. Further in-depth research is needed to determine the value and profitability of implementing Six Sigma and Lean techniques in the healthcare industry.
